# Clinicopathological characteristics, evolution, and treatment outcomes of hormone receptor-negative/HER2-low metastatic breast cancer: a pooled analysis of individual patient data from three prospective clinical trials

**DOI:** 10.3389/fendo.2024.1449278

**Published:** 2024-11-21

**Authors:** Shihui Hu, Yannan Zhao, Yizhao Xie, Shuhui You, Xichun Hu, Jian Zhang, Leiping Wang, Jun Cao, Chengcheng Gong, Biyun Wang

**Affiliations:** ^1^ Department of Medical Oncology, Fudan University Shanghai Cancer Center, Shanghai, China; ^2^ Department of Oncology, Shanghai Medical College, Fudan University, Shanghai, China

**Keywords:** HER2-low, metastatic breast cancer, hormone receptor-negative, chemotherapy, Chinese women, evolution

## Abstract

**Objective:**

With the approval of trastuzumab deruxtecan for the treatment of unresectable/metastatic HER2-low breast cancer, human epidermal growth factor receptor 2 (HER2)-low has emerged as a clinically actionable biomarker. There is an urgent need for a deeper understanding of HER2-low breast cancer patients. Therefore, this study was conducted to explore the clinicopathological characteristics, the evolution of HER2-low status, and its impact on the prognosis of hormone receptor (HoR)-negative/HER2-low metastatic breast cancer (MBC) patients.

**Methods:**

This pooled analysis included 350 metastatic triple-negative breast cancer (mTNBC) patients who received first-line platinum-based chemotherapy at Fudan University Shanghai Cancer Center from November 2007 to July 2022. Patients were categorized into HER2-0 and HER2-low groups based on their HER2 status. Baseline clinicopathological characteristics, evolution of HER2 status between primary and metastatic lesions, and treatment efficacy were compared between the two groups.

**Results:**

Among the 350 mTNBC patients, 34.9% (122/350) were HER2-low and 65.1% (228/350) were HER2-0. Significant differences were observed between HER2-low and HER2-0 patients in terms of age and menopausal status. HER2-low patients were older (54 *vs.* 49 years, *p*=0.002) and had a lower proportion of premenopausal patients (32.8% *vs.* 52.6%, *p*<0.001) compared to HER2-0 patients. No significant differences were observed in progression-free survival (PFS) and overall survival (OS) between HER2-low and HER2-0 patients receiving first-line platinum-based chemotherapy (mPFS: 7.43 *vs.* 8.30 months, *p*=0.389, HR=1.11, 95% CI 0.88-1.40; mOS: 25.37 *vs.* 26.63 months, *p*=0.907, HR=1.02, 95% CI 0.76-1.37). Additionally, 32.3% (41/127) of patients exhibited discordant HER2 status between primary and metastatic lesions, primarily evolving from HER2-0 to HER2-low. Notably, patients with discordant HER2 status had significantly longer PFS compared to those with concordant status (mPFS: 11.07 *vs.* 7.53 months, *p*=0.020). The Cox multivariate analysis showed that HER2 status consistency (*p*=0.026) was an independent predictor of PFS.

**Conclusion:**

In mTNBC patients, those with HER2-low status had similar responses to platinum-based chemotherapy as HER2-0 patients. There was significant discordance in HER2 status between primary and metastatic lesions. Patients with discordant HER2 status had better responses to platinum-based chemotherapy. Therefore, for patients with HER2-0 primary lesions, re-evaluation of HER2 status in metastatic lesions through biopsy may offer new treatment opportunities.

## Introduction

Breast cancer, the second most common cancer globally, accounts for 2.3 million new cases annually, representing 11.6% of all new cancer cases (1). Among women, breast cancer is the most prevalent cancer and a leading cause of cancer-related deaths (1). The human epidermal growth factor receptor 2 (HER2) belongs to the epidermal growth factor receptor family and plays a crucial role in the biological behavior and pathogenesis of breast cancer (2). Approximately 15%-20% of breast cancers exhibit HER2 amplification or overexpression (3), and the development of anti-HER2 therapies has significantly improved treatment outcomes for HER2-positive breast cancer (2, 4–6).

According to the 2023 European Society for Medical Oncology (ESMO) expert consensus statements (7), HER2-low expression is defined as an immunohistochemistry (IHC) score of 1+ or 2+/*in situ* hybridization (ISH) not amplified, accounting for 45%-55% of all breast cancer types (8). Currently, HER2 status is primarily determined using IHC and ISH (9). Real-time fluorescent quantitative polymerase chain reaction (RTFQ-PCR) is also a rapid, sensitive, and high-throughput technique for detecting HER2 gene amplification (10). Despite clinical practice traditionally categorizing HER2 status as positive (IHC 3+ or IHC 2+/ISH-positive) or negative (IHC 0, IHC 1+, IHC 2+/ISH-negative) (9), studies have found that breast cancers with IHC 0 and IHC 1+ differ not only in HER2 protein expression levels but also in estrogen receptor (ER) status, primary tumor size, lymph node involvement, pathologic complete response (pCR) rates after neoadjuvant therapy, and disease-free survival (DFS) (11–13). This suggests that HER2-low breast cancer may have distinct molecular characteristics (8, 12, 14, 15), which are not yet fully understood.

In clinical trials targeting early-stage HER2-low breast cancer, the NSABP B-31 and N9831 studies indicated potential benefits of adjuvant trastuzumab therapy for breast cancer patients (16, 17). However, the phase III prospective randomized controlled study (NSABP B-47) showed that adding trastuzumab to adjuvant chemotherapy did not improve invasive disease-free survival (iDFS), 5-year distant recurrence-free interval, or overall survival (OS) in HER2-low breast cancer patients (10). A pooled analysis from four prospective neoadjuvant clinical trials involving 1098 HER2-low and 1212 HER2-negative primary breast cancer patients reported that the pCR rate was significantly lower in the HER2-low group compared to the HER2-negative group (29.2% *vs.* 39.0%, *p*=0.0002), long-term outcomes, however, were significantly better in HER2-low patients (3-year DFS: 83.4% *vs.* 76.1%, *p*=0.0084; 3-year OS: 91.6% *vs.* 85.8%, *p*=0.0016) (18).

In recent years, numerous clinical trials targeting advanced HER2-low breast cancer have emerged. The single-arm phase II studies, TDM4258g, and 4374g, initially suggested the sensitivity of HER2-low breast cancer to T-DM1, but conclusions remain unclear due to the small patient number (19, 20). The phase III DESTINY-Breast04 study, the first to focus on HER2-low breast cancer and yield positive results, demonstrated that DS-8201a significantly prolonged progression-free survival (PFS) and OS in HER2-low metastatic breast cancer (MBC) patients compared to the physician’s choice of chemotherapy, regardless of hormone receptor (HoR) status (HoR+: mPFS: 10.1 *vs.* 5.4 months, *p*<0.001; mOS: 23.9 *vs.* 17.5 months, *p*=0.003; HoR-: mPFS: 8.5 *vs.* 2.9 months; mOS: 18.2 *vs.* 8.3 months) (21). This confirmed the efficacy of DS-8201a in HER2-low MBC. In April 2022, DS-8201a received Breakthrough Therapy Designation (BTD) from the US Food and Drug Administration (FDA) for the treatment of adult patients with unresectable or metastatic HER2-low breast cancer (22). However, the prognostic value of HER2-low expression remains controversial. While previous studies have explored the prognosis of HER2-low MBC patients, results have been inconsistent (23–25).

Several prospective clinical trials have been conducted in metastatic triple-negative breast cancer (mTNBC) at Fudan University Shanghai Cancer Center (FUSCC) (26–28). We conducted a pooled analysis of the individual data of 350 mTNBC patients from three clinical trials to analyze the clinicopathological characteristics of HER2-low and HER2-0 patients, the evolution of HER2 status between primary and metastatic sites, and the efficacy of first-line platinum-based chemotherapy regimens.

## Patients and methods

### Patients

This study included 350 patients with recurrent or metastatic triple-negative breast cancer from three previous prospective clinical studies conducted at FUSCC: NCT00601159 (GP) (26), NCT02546934 (GAP) (28), and NCT02341911 (GPGC). In these three prospective studies, researchers recruited mTNBC patients who had not received prior chemotherapy to evaluate the efficacy and safety of various platinum-based regimens as first-line treatments for mTNBC (**Supplementary Table S1**). Although the patient list was derived from prospective trials, clinical and pathological data for each patient were retrospectively re-confirmed and collected in this study. Data were directly collected from patients’ electronic medical records. The inclusion criteria for this study were as follows: (1) female; (2) aged over 18 years; (3) diagnosed with stage IV or recurrent breast cancer by histological or cytological methods; (4) diagnosed with TNBC, with a detailed pathological report documenting HER2 status. ER, PR, and HER2 status were determined locally by IHC of patients’ primary or metastatic tumor sections. ER-negative and PR-negative status was defined as <1% staining in the nuclei by IHC. HER2-negative status was determined by IHC staining 0 to 1+ or fluorescence *in situ* hybridization (FISH) ratio <2.0 if IHC 2+ or IHC not performed; (5) having received at least one cycle of chemotherapy during advanced systemic treatment; and (6) having complete medical records. All patients participating in the clinical trials signed informed consent forms, agreeing to the use of their clinical data for subsequent analyses.

Patients in this study were divided into HER2-0 and HER2-low groups based on HER2 status, as defined in **Supplementary Table S2** (14). For patients with both primary and metastatic lesion pathology results, if a discrepancy in HER2 status was observed between the primary and metastatic lesions, the HER2 status was determined based on the pathological result from the metastatic lesion. If multiple metastatic sites were biopsied and showed inconsistent HER2 results, the predominant HER2 expression pattern was used. In cases where no predominant pattern was identified, the HER2 status was determined by the biopsy result from the largest metastatic lesion. Additionally, changes in HER2 expression between primary and metastatic lesions were documented.

### Evaluation

Baseline clinicopathological characteristics, including age, menopausal status, tumor grade, histological type, clinical stage at diagnosis, Ki-67, disease-free interval (DFI), number of metastatic sites, and metastatic sites, were compared between the HER2-low and HER2-0 groups. DFI was defined as the time from the initial breast cancer diagnosis to the first relapse. Visceral metastasis was defined as the involvement of internal organs, including lung, liver, peritoneum, or pleural and central nervous system recurrences.

The primary endpoint was PFS, and the secondary endpoint was OS. PFS was defined as the time from the start of advanced first-line chemotherapy to disease progression or death due to any cause. OS was defined as the time from the start of advanced first-line chemotherapy to death due to any causes or the last follow-up visit. Complete response (CR), partial response (PR), stable disease (SD), and progression disease (PD) were assessed according to Response Evaluation Criteria in Solid Tumors (RECIST) version 1.1.

### Statistical analysis

Categorical variables were expressed as frequencies (n) and percentages (%), while continuous variables were presented as medians and ranges. Baseline characteristics between the HER2-0 and HER2-low groups were compared using the chi-square or Fisher’s exact test for categorical variables, and the Wilcoxon rank-sum test for continuous variables. The concordance of HER2 expression between the primary tumor and the corresponding metastatic tumor was assessed using Cohen’s kappa coefficient (K). Kaplan-Meier curves were used to estimate median PFS and OS, along with their corresponding 95% confidence intervals (CIs), with comparisons between groups performed using the log-rank test. Stepwise multivariate Cox proportional hazards models evaluated potential predictors of treatment efficacy, with effects quantified as hazard ratios (HRs), alongside their 95% CIs and *p*-values. The proportional hazards assumption in the Cox model was assessed using the Schoenfeld residuals test, and no violations were observed. Variables with a *p*-value below 0.1 in univariate analysis were included in multivariate analysis. Sankey diagrams illustrated the evolution of HER2 status between primary and metastatic lesions. Statistical analyses were performed using SPSS software (version 21.0). *p*<0.05 was considered statistically significant.

## Result

### Patient characteristics

This study included 350 patients who were diagnosed with mTNBC between November 2007 and July 2022 (**Table 1**). Among them, 228 patients (65.1%) were categorized as HER2-0 MBC, while 122 patients (34.9%) as HER2-low. Significant differences were observed in age and menopausal status between HER2-low and HER2-0 patients. The median age of patients in the HER2-low group was significantly higher than that of the HER2-0 group (54 *vs.* 49 years, *p*=0.002). In the HER2-low group, 32.8% of patients were premenopausal, compared to 52.6% in the HER2-0 group, indicating a lower proportion of premenopausal patients in the HER2-low group (*p*<0.001). Furthermore, in the HER2-low group, the proportion of patients who experienced recurrence or metastasis within two years of the initial breast cancer diagnosis was relatively lower compared to the HER2-0 group (45.9% *vs.* 60.5%, *p*=0.055). Among HER2-low patients, 50.8% had grade III tumors, and 93.4% had invasive ductal carcinoma (IDC). Additionally, 32.8% of HER2-low patients had ≥3 metastatic sites, and 62.3% had visceral metastases (**Table 1**).

**Table 1:** Baseline clinicopathologic characteristics of patients stratified by HER2 status (HER2-low vs. HER2-0).

Characteristic	HER2-low N=122 (%)	HER2-0 N=228 (%)	Total N=350 (%)	*p*-value
Age				
Median (range)	54 (23-75)	49 (22-72)	50 (22-75)	0.002*
<50 years	46 (37.7)	123 (53.9)	169 (48.3)	0.004*
≥50 years	76 (62.3)	105 (46.1)	181 (51.7)	
Menopausal status				<0.001*
Premenopause	40 (32.8)	120 (52.6)	160 (45.7)	
Postmenopause	82 (67.2)	108 (47.4)	190 (54.3)	
Grade				0.114
II	22 (18.0)	33 (14.5)	55 (15.7)	
III	62 (50.8)	142 (62.3)	204 (58.3)	
Unknown	38 (31.1)	53 (23.2)	91 (26.0)	
Histological type^b^				0.642
IDC	114 (93.4)	214 (93.9)	328 (93.7)	
ILC	1 (0.8)	1 (0.4)	2 (0.6)	
Other	5 (4.1)	12 (5.3)	17 (4.9)	
Unknown	2 (1.6)	1 (0.4)	3 (0.9)	
Stage at diagnosis				0.203
I	12 (9.8)	42 (18.4)	54 (15.4)	
II	54 (44.3)	102 (44.7)	156 (44.6)	
III	32 (26.2)	52 (22.8)	84 (24.0)	
IV	17 (13.9)	21 (9.2)	38 (10.9)	
Unknown	7 (5.7)	11 (4.8)	18 (5.1)	
Ki-67				
Median (range)	50% (5%-95%)	50% (5%-90%)	50% (5%-95%)	0.473
≤15%	7 (7.0)	11 (5.7)	18 (6.1)	0.750
15.1%-35.0%	23 (23.0)	39 (20.2)	62 (21.2)	
>35.0%	70 (70.0)	143 (74.1)	213 (72.7)	
DFI^c^				0.055
<2 years	56 (45.9)	138 (60.5)	194 (55.4)	
2 to 5 years	41 (33.6)	61 (26.8)	102 (29.1)	
>5 years	8 (6.6)	8 (3.5)	16 (4.6)	
*De novo* stage IV	17 (13.9)	21 (9.2)	38 (10.9)	
Number of metastatic sites^d^				0.494
1	44 (36.1)	89 (39.0)	133 (38.0)	
2	38 (31.1)	78 (34.2)	116 (33.1)	
≥3	40 (32.8)	61 (26.8)	101 (28.9)	
Metastatic sites				
Liver	29 (23.8)	59 (25.9)	88 (25.1)	0.665
Lung	49 (40.2)	104 (45.6)	153 (43.7)	0.327
Bone	44 (36.1)	68 (29.8)	112 (32.0)	0.233
Brain	4 (3.3)	4 (1.8)	8 (2.3)	0.457
Visceral	76 (62.3)	151 (66.2)	227 (64.9)	0.463
First-line chemotherapy^e^				0.709
GP	67 (54.9)	134 (58.8)	201 (57.4)	
AP	29 (23.8)	46 (20.2)	75 (21.4)	
GC	26 (21.3)	48 (21.1)	74 (21.1)	

*p<0.05.

a. %, the percentage of patients in each category relative to the total number of patients in each group (HER2-low, HER2-0, and Total).

b. IDC, invasive ductal carcinoma; ILC, invasive lobular carcinoma.

c. Disease-free interval (DFI) is defined as the time from diagnosis of breast cancer to first relapse.

d. Data of Metastases were collected at the time before the 1^st^ line chemotherapy for MBC.

e. GP, Gemcitabine combined with Cisplatin; AP, Nab-paclitaxel combined with Cisplatin; GC, Gemcitabine combined with Carboplatin.

In this study, 54.9% (67/122) of HER2-low patients received gemcitabine combined with cisplatin (GP) as first-line chemotherapy, 23.8% (29/122) received nab-paclitaxel combined with cisplatin (AP), and 21.3% (26/122) received gemcitabine combined with carboplatin (GC). There was no significant difference in the distribution of platinum-based regimens between the HER2-low and HER2-0 groups (*p*=0.709) (**Table 1**).

### Distribution and evolution of HER2 status in primary and metastatic lesions

**Figure 1:**
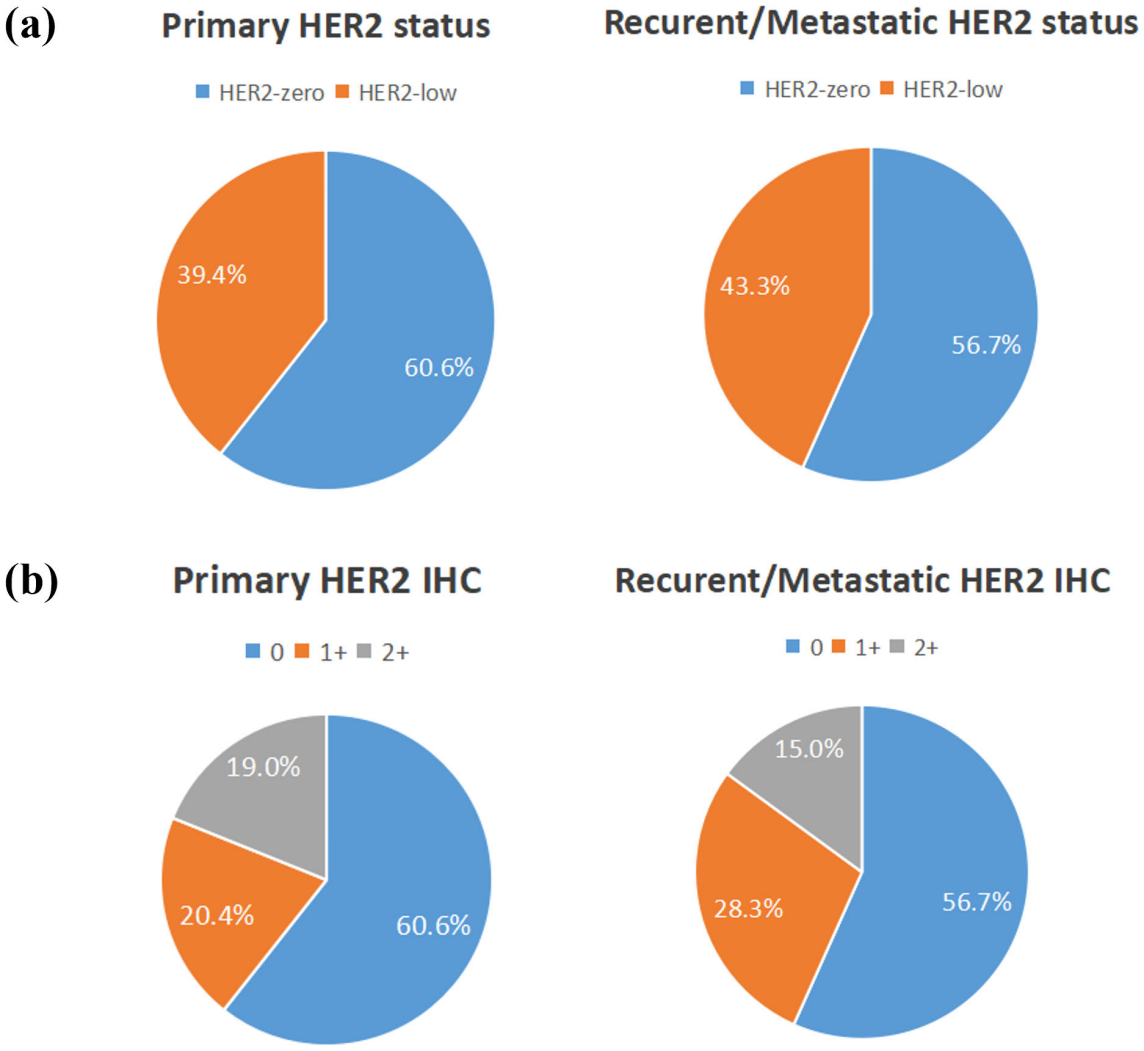
The compositions of human epidermal growth factor receptor 2 (HER2)-negative population by **(A)** HER2 status and **(B)** HER2 immunohistochemistry (IHC) score.

**Figure 2:**
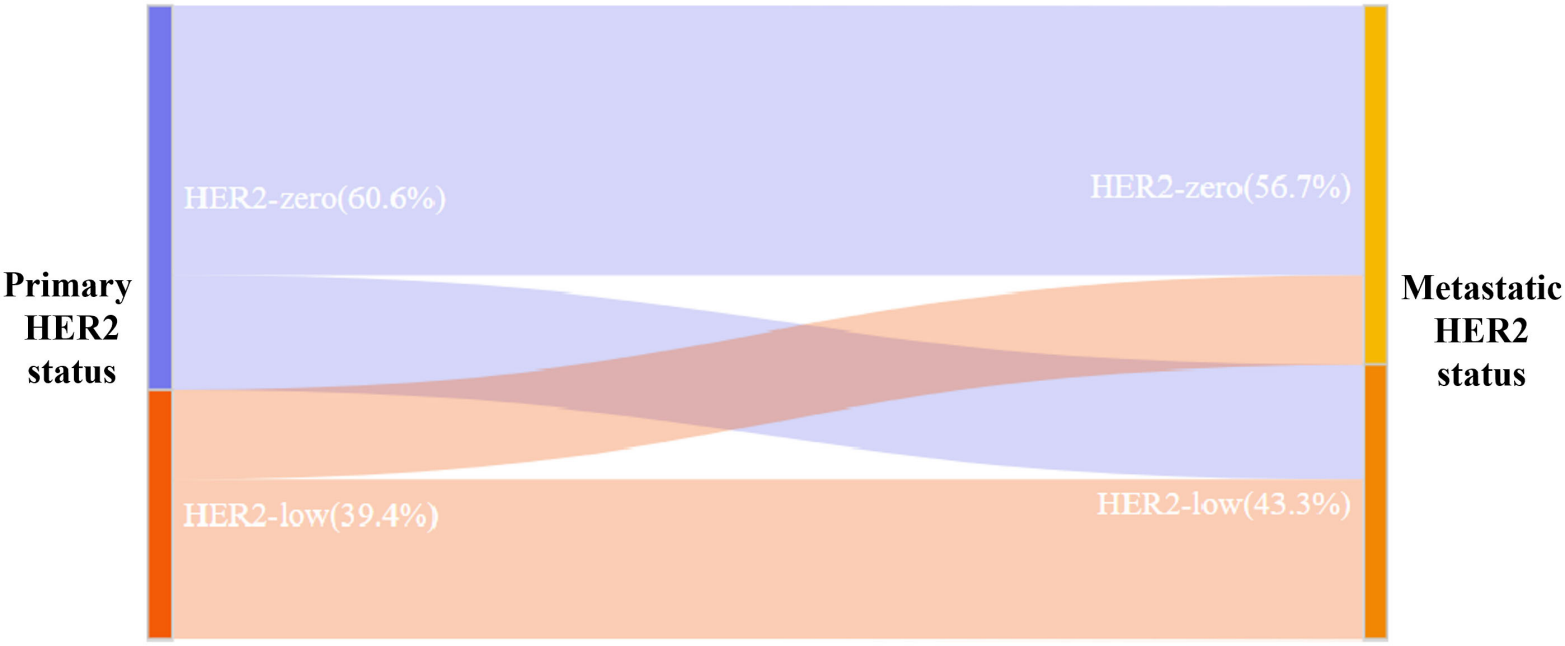
Sankey diagram of HER2 status evolution between primary and metastatic breast cancer.

**Figure 3:**
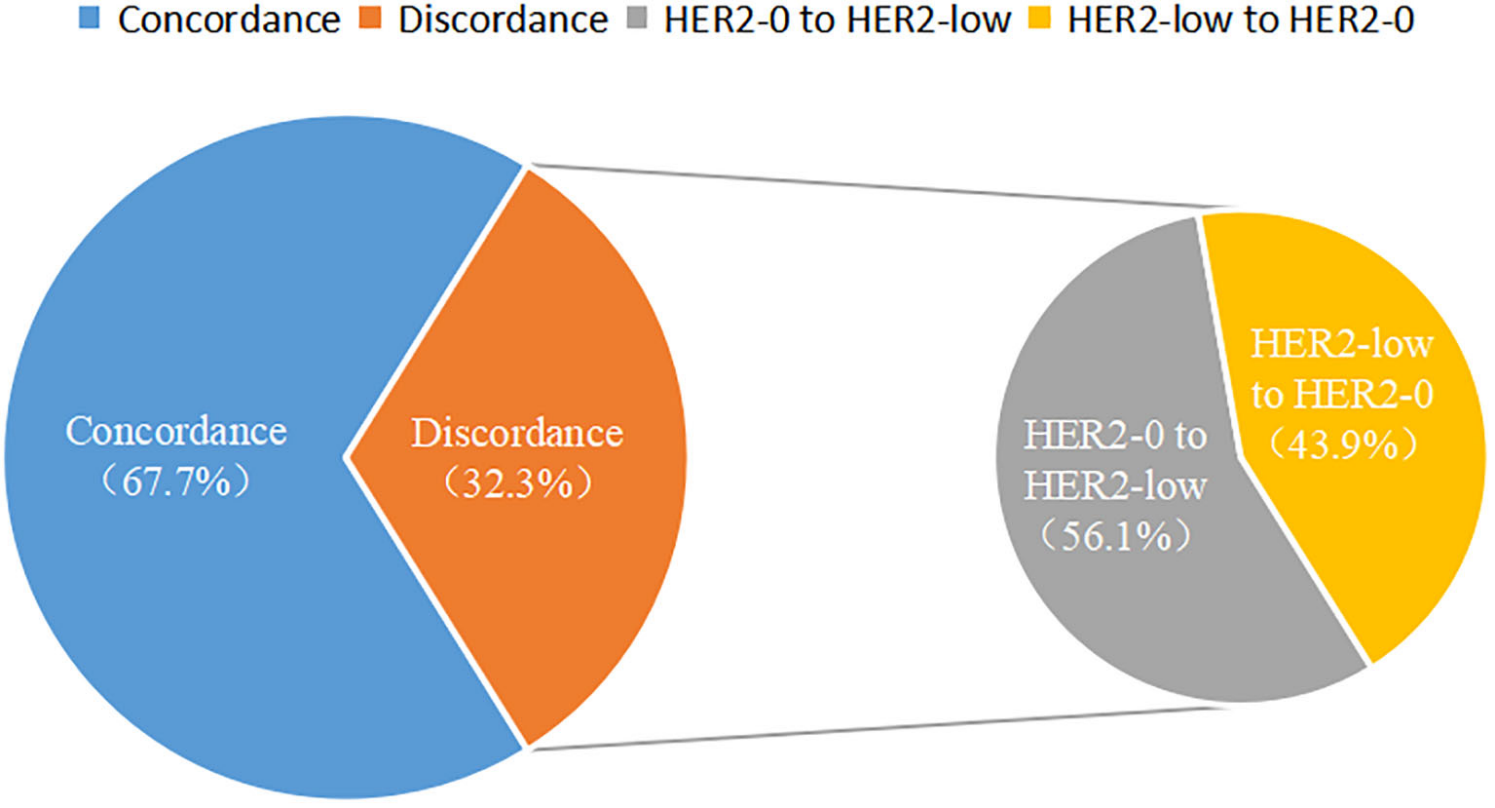
Pie chart of the consistency of HER2 status between primary and metastatic breast cancer.

Among the 350 mTNBC patients included in this study, 36.3% (127/350) had pathological information for both primary and metastatic lesions. Of these 127 patients, 39.4% (50/127) of the primary lesions were HER2-low, and 43.3% (55/127) of the metastatic lesions were HER2-low (**Figure 1A**, **Table 2**). HER2 status was concordant between primary and metastatic lesions in 67.7% (86/127) of patients, while 32.3% (41/127) showed discordant HER2 status (K=0.34, 95% CI 0.17-0.50, *p*<0.001). Among the discordant patients, 56.1% (23/41) transitioned from HER2-0 to HER2-low, and 43.9% (18/41) transitioned from HER2-low to HER2-0 (**Figures 2**, **3**, **Table 2**). According to the HER2 IHC score, 55.9% (71/127) of patients had concordant HER2 status, while 44.1% (56/127) had discordant HER2 status (K=0.23, 95% CI 0.09-0.36, *p*<0.001) (**Table 3**). The proportion of HER2 1+ patients increased from 20.4% (26/127) in primary lesions to 28.3% (36/127) in metastatic lesions (**Figure 1B**, **Table 3**).

**Table 2:** HER2 expression evolution from primary tumors to recurrence/metastasis according to HER2 status (HER2-low *vs.* HER2-0).

		Recurrence/Metastasis N (%)		Total
		HER2-0	HER2-low	
Primary N (%)	HER2-0	54 (42.5)	23 (18.1)	77 (60.6)
	HER2-low	18 (14.2)	32 (25.2)	50 (39.4)
Total		72 (56.7)	55 (43.3)	127 (100.0)

HER2 status discordance rate=32.3% (K=0.34, 95% CI 0.17-0.50, *p*<0.001).

**Table 3:** HER2 expression evolution from primary tumors to recurrence/metastasis according to HER2 IHC score (HER2 0 *vs.* HER2 1+ *vs.* HER2 2+).

		Recurrence/Metastasis N (%)			Total
		HER2 0	HER2 1+	HER2 2+	
Primary N (%)	HER2 0	54 (42.5)	19 (15.0)	4 (3.1)	77 (60.6)
	HER2 1+	16 (12.6)	6 (4.7)	4 (3.1)	26 (20.4)
	HER2 2+	2 (1.6)	11 (8.7)	11 (8.7)	24 (19.0)
Total		72 (56.7)	36 (28.3)	19 (15.0)	127 (100.0)

HER2 status discordance rate=44.1% (K=0.23, 95% CI 0.09-0.36, *p*<0.001).

**Table 4** compared the baseline clinicopathological characteristics of patients with concordant (n=86) and discordant (n=41) HER2 status between primary and metastatic lesions. It was found that patients with discordant HER2 status were relatively older (≥50 years: 56.1% *vs.* 53.5%) and had a higher proportion of postmenopausal patients (68.3% *vs.* 58.1%). They also had a relatively lower proportion of patients with visceral metastases (56.1% *vs.* 67.4%), although these differences were not statistically significant. Overall, the baseline clinicopathological characteristics were comparable between the two groups.

**Table 4:** Comparison of baseline characteristics between patients with concordant HER2 states and discordant HER2 status.

	HER2 status		Tatal (%)	*p-*values
	Discordant (%)	Concordant (%)		
Age				0.783
<50 years	18 (43.9)	40 (46.5)	58 (45.7)	
≥50 years	23 (56.1)	46 (53.5)	69 (54.3)	
Menopausal status				0.272
Premenopause	13 (31.7)	36 (41.9)	49 (38.6)	
Postmenopause	28 (68.3)	50 (58.1)	78 (61.4)	
Grade				0.360
II	6 (14.6)	16 (18.6)	22 (17.3)	
III	20 (48.8)	49 (57.0)	69 (54.3)	
Unknown	15 (36.6)	21 (24.4)	36 (28.3)	
Histological type^a^				0.349
IDC	35 (85.4)	80 (93.0)	115 (90.6)	
ILC	1 (2.4)	1 (1.2)	2 (1.6)	
Other	4 (9.8)	5 (5.8)	9 (7.1)	
Unknown	1 (2.4)	0 (0.0)	1 (0.8)	
Stage at diagnosis				0.732
I	10 (24.4)	14 (16.3)	24 (18.9)	
II	19 (46.3)	41 (47.7)	60 (47.2)	
III	5 (12.2)	17 (19.8)	22 (17.3)	
IV	3 (7.3)	7 (8.1)	10 (7.9)	
Unknown	4 (9.8)	7 (8.1)	11 (8.7)	
Ki-67				0.274
≤15%	5 (12.5)	4 (4.7)	9 (7.2)	
15.1%-35.0%	8 (20.0)	21 (24.7)	29 (23.2)	
>35.0%	27 (67.5)	60 (70.6)	87 (69.6)	
DFI^b^				0.957
<2 years	20 (48.8)	42 (48.8)	62 (48.8)	
2 to 5 years	14 (34.1)	31 (36.0)	45 (35.4)	
>5 years	4 (9.8)	6 (7.0)	10 (7.9)	
*De novo* stage IV	3 (7.3)	7 (8.1)	10 (7.9)	
Number of metastatic sites^c^				0.229
1	18 (43.9)	30 (34.9)	48 (37.8)	
2	16 (39.0)	29 (33.7)	45 (35.4)	
≥3	7 (17.1)	27 (31.4)	34 (26.8)	
Metastatic sites				
Liver	9 (22.0)	23 (26.7)	32 (25.2)	0.561
Lung	16 (39.0)	35 (40.7)	51 (40.2)	0.857
Bone	12 (29.3)	27 (31.4)	39 (30.7)	0.808
Brain	1 (2.4)	2 (2.3)	3 (2.4)	1.000
Visceral	23 (56.1)	58 (67.4)	81 (63.8)	0.214
First-line chemotherapy^d^				0.286
GP	23 (56.1)	41 (47.7)	64 (50.4)	
AP	11 (26.8)	19 (22.1)	30 (23.6)	
GC	7 (17.1)	26 (30.2)	33 (26.0)	

a. IDC, invasive ductal carcinoma; ILC, invasive lobular carcinoma.

b. Disease-free interval (DFI) is defined as the time from diagnosis of breast cancer to first relapse.

c. Data of Metastases were collected at the time before the 1^st^ line chemotherapy for MBC.

d. GP, Gemcitabine combined with Cisplatin; AP, Nab-paclitaxel combined with Cisplatin; GC, Gemcitabine combined with Carboplatin.

### Efficacy

**Figure 4:**
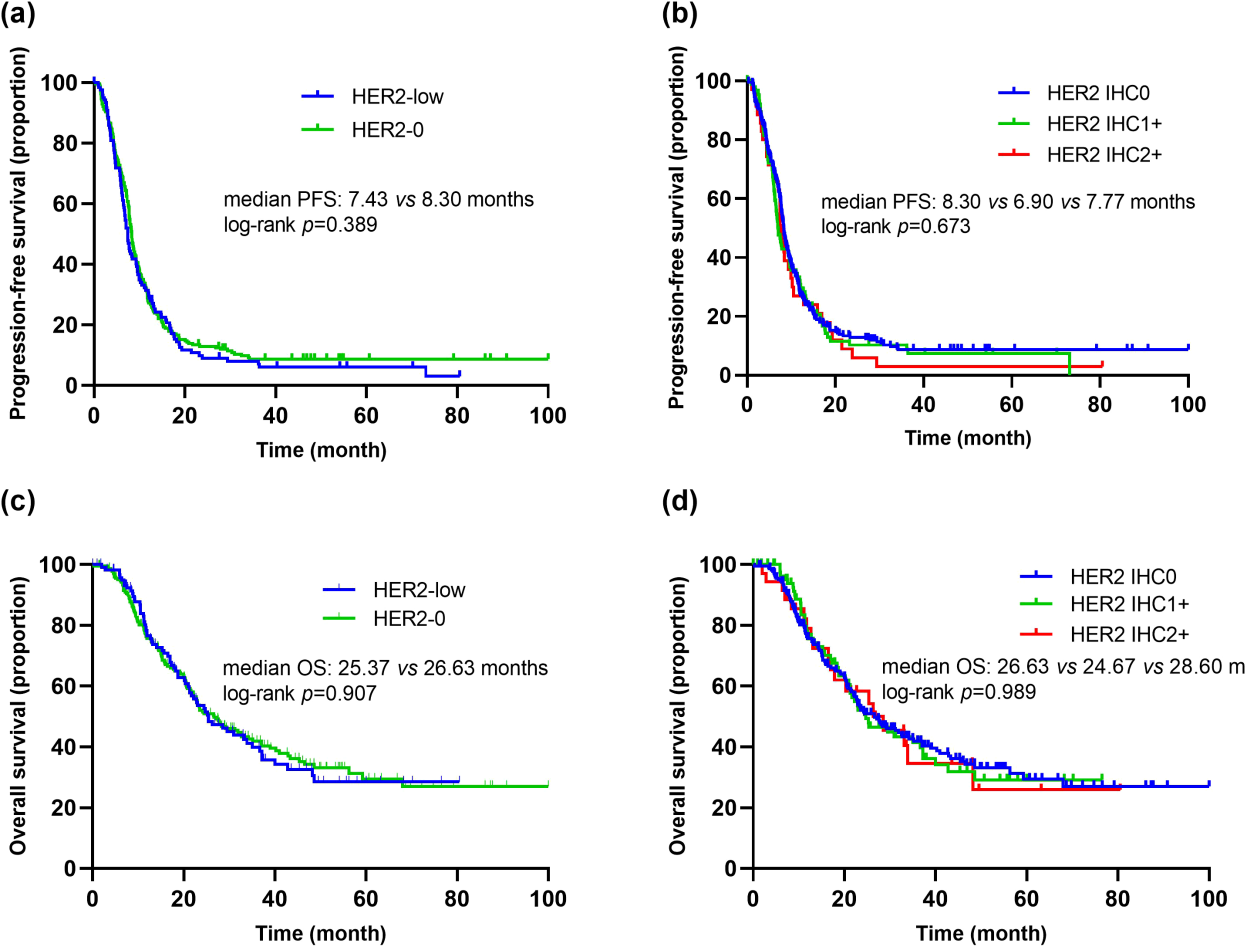
Kaplan–Meier curves of PFS and OS in the first-line platinum-based chemotherapy. **(A)** Comparison of PFS between patients with HER2-low and HER2-0. **(B)** Comparison of PFS between patients with HER2 IHC 0, IHC 1+ and IHC 2+. **(C)** Comparison of OS between patients with HER2-low and HER2-0. **(D)** Comparison of OS between patients with HER2 IHC 0, IHC 1+ and IHC 2+.

As of August 2023, the median follow-up time was 63.4 months (interquartile range [IQR], 50.3-76.5 months). Among the patients who received first-line platinum-based chemotherapy, 91.0% (111/122) of the HER2-low group experienced PFS events, with a median PFS of 7.43 months (95% CI 6.62-8.24). In the HER2-0 group, 88.6% (202/228) experienced PFS events, with a median PFS of 8.30 months (95% CI 7.75-8.85) (**Figure 4A**). Kaplan-Meier survival analysis showed no significant difference in PFS between HER2-low and HER2-0 patients receiving first-line platinum-based chemotherapy (*p*=0.389, HR=1.11, 95% CI 0.88-1.40) (**Figure 4A**). Furthermore, there were no significant differences in PFS among patients with different HER2 IHC scores (0, 1+, 2+) (mPFS: 8.30 *vs.* 6.90 *vs.* 7.77 months, *p*=0.673) (**Figure 4B**).

Additionally, among patients receiving first-line platinum-based chemotherapy, 55.7% (68/122) of the HER2-low group died, with a median OS of 25.37 months (95% CI 18.26-32.49). In the HER2-0 group, 55.3% (126/228) died, with a median OS of 26.63 months (95% CI 20.61-32.65). There was no significant difference in median OS between the two groups (*p*=0.907, HR=1.02, 95% CI 0.76-1.37) (**Figure 4C**). Similarly, there were no significant differences in OS among patients with different HER2 IHC scores (0, 1+, 2+) (mOS: 26.63 *vs.* 24.67 *vs.* 28.60 months, *p*=0.989) (**Figure 4D**).

Among patients receiving GP as first-line chemotherapy, there were no significant differences in PFS and OS between the HER2-low and HER2-0 groups (mPFS: 6.70 *vs.* 8.07 months, *p*=0.418, HR=1.14, 95% CI 0.83-1.55; mOS: 25.37 *vs.* 21.37 months, *p*=0.452, HR=0.86, 95% CI 0.59-1.27) (**Supplementary Figures S1A, B**). Similarly, there were no significant differences in PFS and OS between HER2-low and HER2-0 groups receiving AP or GC as first-line chemotherapy (AP: mPFS: 9.63 *vs.* 9.33 months, *p*=0.692, HR=0.90, 95% CI 0.55-1.49; mOS: 33.87 months *vs.* not reached, *p*=0.275, HR=1.54, 95% CI 0.70-3.39; GC: mPFS: 6.57 *vs.* 7.67 months, *p*=0.170, HR=1.43, 95% CI 0.86-2.38; mOS: 18.70 *vs.* 27.60 months, *p*=0.250, HR=1.41, 95% CI 0.78-2.52) (**Supplementary Figures S1C–F**).

**Figure 5:**
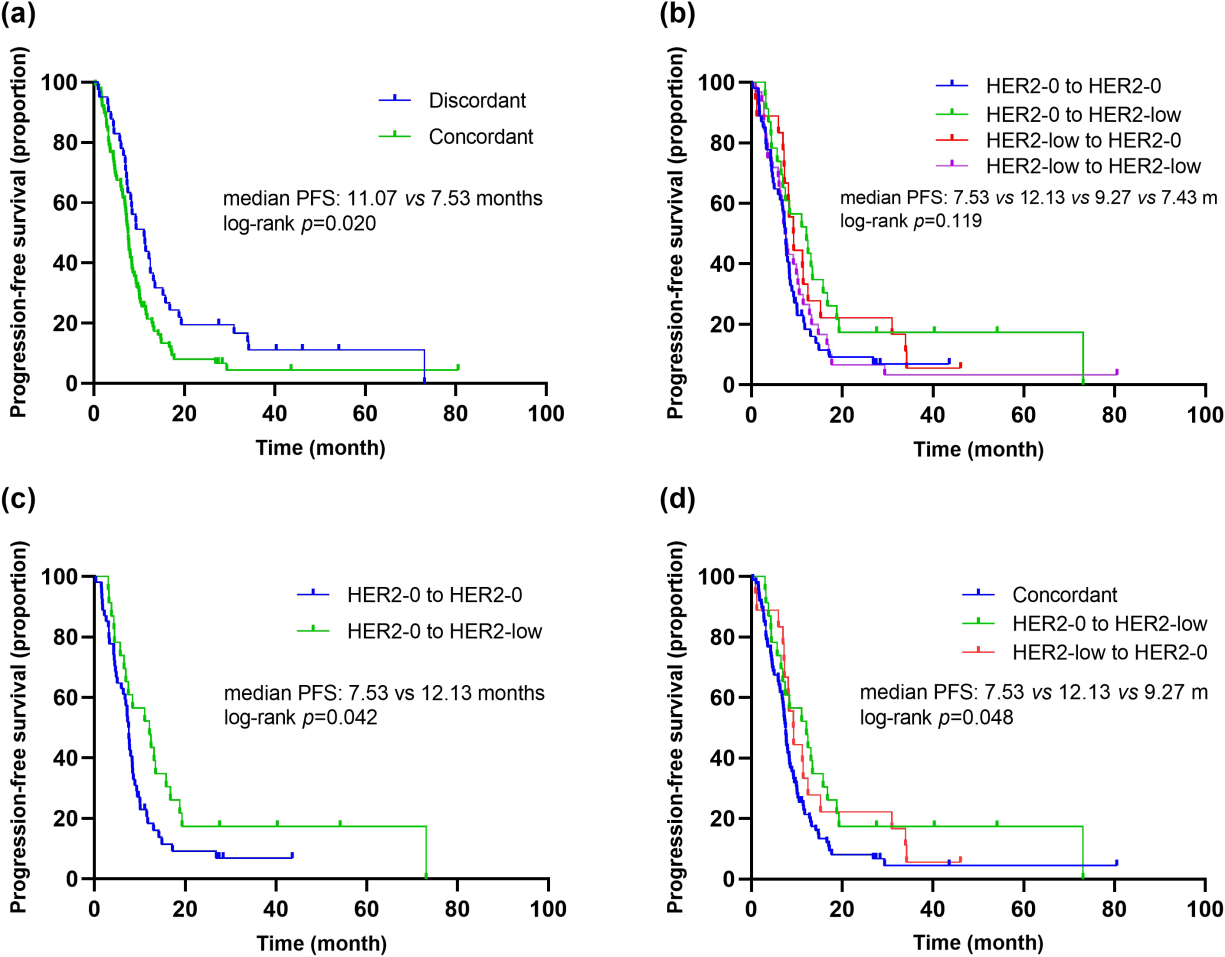
Kaplan–Meier curves of PFS in the first-line platinum-based chemotherapy. **(A)** Comparison of PFS in patients with concordant *vs.* discordant HER2 status between primary and metastasis breast cancer. **(B)** Comparison of PFS in patients with different HER2 status transitions (HER2-0 to HER2-0 *vs.* HER2-0 to HER2-low *vs.* HER2-low to HER2-0 *vs.* HER2-low to HER2-low). **(C)** Comparison of PFS in patients with different HER2 status transitions (HER2-0 to HER2-0 *vs.* HER2-0 to HER2-low). **(D)** Comparison of PFS in patients with different HER2 status transitions (concordant HER2 status *vs.* HER2-0 to HER2-low *vs.* HER2-low to HER2-0).

Additionally, we evaluated the impact of HER2 status consistency between primary and metastatic lesions on the efficacy of first-line platinum-based chemotherapy. The results showed that patients with discordant HER2 status between primary and metastatic lesions had significantly longer PFS compared to those with concordant HER2 status (mPFS: 11.07 *vs.* 7.53 months, *p*=0.020, HR=1.60, 95% CI 1.07-2.39) (**Figure 5A**). Further pairwise comparisons based on HER2 status evolution indicated that patients transitioning from HER2-0 to HER2-low had significantly longer PFS compared to those with concordant HER2-0 status (12.13 *vs.* 7.53 months, *p*=0.042, HR=0.57, 95% CI 0.33-0.99) (**Figures 5B, C**). Moreover, when comparing PFS among patients with concordant HER2 status, those with a transition from HER2-0 to HER2-low, and those with a transition from HER2-low to HER2-0, there was a significant difference in PFS across the three groups (mPFS: 7.53 *vs.* 12.13 *vs.* 9.27 months, *p*=0.048) (**Figure 5D**). Although differences in PFS were observed among the various HER2 evolution patterns (HER2-0 to HER2-0 *vs.* HER2-low to HER2-low, HER2-0 to HER2-0 *vs.* HER2-low to HER2-0, HER2-0 to HER2-low *vs.* HER2-low to HER2-0, HER2-low to HER2-0 *vs.* HER2-low to HER2-low), these differences did not reach statistical significance (**Supplementary Figure S2**).

The analysis of the impact of HER2 status consistency on OS revealed that patients with discordant HER2 status had a relatively longer OS compared to those with concordant HER2 status (mOS: 46.53 *vs.* 27.77 months, *p*=0.054) (**Supplementary Figure S3A**). Further comparison of different HER2 status evolution patterns demonstrated significant differences in OS among patients with the following evolution patterns: HER2-0 to HER2-0 *vs.* HER2-0 to HER2-low *vs.* HER2-low to HER2-0 *vs.* HER2-low to HER2-low (mOS: 23.50 *vs.* 26.33 *vs.* 46.53 *vs.* 33.13 months, *p*=0.046) (**Supplementary Figure S3B**).

Univariate and multivariate COX regression analyses were conducted to identify potential factors influencing PFS in mTNBC patients receiving first-line platinum-based chemotherapy. Univariate analysis showed that the number of metastatic sites, liver metastasis, bone metastasis, and HER2 status consistency were significantly associated with PFS (**Table 5**). Subsequent multivariate analysis indicated that HER2 status consistency was an independent predictor of PFS. Patients with discordant HER2 status between primary and metastatic lesions had significantly longer PFS compared to those with concordant HER2 status (11.07 *vs.* 7.53 months, *p*=0.020, HR=1.58, 95% CI 1.06-2.36) (**Table 5**). Additionally, univariate analysis of OS indicated that the number of metastatic sites and bone metastasis were significantly associated with OS. Multivariate analysis indicated that the number of metastatic sites was an independent predictor of OS (**Table 5**).

**Table 5:** Univariate and multivariate Cox regression analysis of factors associated with PFS and OS of the first-line chemotherapy.

	PFS				OS			
	Univariate analysis		Multivariate analysis		Univariate analysis		Multivariate analysis	
	HR (95% CI)	*p*- value	HR (95% CI)	*p*- value	HR (95% CI)	*p*- value	HR (95% CI)	*p*- value
Age (<50 *vs* ≥50 years)	0.95 (0.76-1.19)	0.665			0.94 (0.71-1.25)	0.664		
Menopausal status (premenopausal *vs* postmenopause)	1.01 (0.81-1.26)	0.925			0.96 (0.72-1.28)	0.785		
Grade (III *vs* II)	0.87 (0.63-1.19)	0.381			1.05 (0.71-1.56)	0.797		
Ki-67	0.81 (0.49-1.34)	0.415			0.84 (0.43-1.62)	0.598		
*De novo* stage IV (yes *vs* no)	0.80 (0.56-1.15)	0.232			0.63 (0.39-1.03)	0.065	1.05 (0.43-2.53)	0.916
Number of metastatic sites (≥3 *vs* <3)	1.56 (1.23-1.97)	0.001*	1.45 (0.93-2.25)	0.102	2.09 (1.54-2.84)	0.001*	2.22 (1.20-4.10)	0.011*
Liver metastasis (yes *vs* no)	1.52 (1.17-1.96)	0.002*	1.37 (0.86-2.19)	0.188	1.34 (0.97-1.85)	0.080	1.04 (0.55-1.95)	0.905
Lung metastasis (yes *vs* no)	1.08 (0.86-1.34)	0.522			1.24 (0.93-1.64)	0.141		
Bone metastasis (yes *vs* no)	1.34 (1.06-1.69)	0.015*	0.93 (0.60-1.44)	0.736	1.63 (1.21-2.19)	0.001*	1.32 (0.75-2.32)	0.343
Brain metastasis (yes *vs* no)	0.87 (0.39-1.96)	0.745			1.31 (0.49-3.53)	0.596		
Visceral metastasis (yes *vs* no)	1.19 (0.94-1.50)	0.146			1.26 (0.94-1.70)	0.123		
HER2 status (HER2-low *vs* HER2-0)	1.11 (0.88-1.40)	0.390			1.02 (0.76-1.37)	0.907		
HER2 status evolution (concordance *vs* discordance)	1.60 (1.07-2.39)	0.021*	1.58 (1.06-2.36)	0.026*	1.70 (0.99-2.94)	0.057	1.62 (0.93-2.82)	0.088

*p<0.05.

## Discussion

This study included 350 mTNBC patients from three clinical trials and explored their clinicopathological characteristics, the evolution of HER2 status between primary and metastatic lesions, and treatment efficacy of first-line platinum-based chemotherapy to compare differences between HER2-0 and HER2-low patients. We found that HER2 status did not significantly impact the efficacy of first-line platinum-based chemotherapy in advanced stages. However, patients with discordant HER2 status between primary and metastatic lesions had significantly better efficacy from first-line platinum-based chemotherapy compared to those with concordant HER2 status.

In the design of this study, we selected mTNBC patients from prospective clinical trials based on the following considerations. First, accurate assessment of HER2 status is crucial for comparing the clinicopathological characteristics and chemotherapy efficacy between HER2-0 and HER2-low patients. In these prospective studies, most patients’ HER2 status was evaluated by experienced pathologists at our center. For patients tested at other hospitals, re-evaluation was conducted at our center after enrollment. Choosing patients from clinical trials effectively reduced inter-laboratory discrepancies and ensured consistency in HER2 status assessment. Second, all patients from these prospective studies received first-line treatment at our center and were hospitalized, ensuring the completeness of electronic medical records, standardization of treatment regimens, and timely efficacy assessment. This patient selection approach better reflected the actual clinical conditions of HER2-low patients.

Among the mTNBC patients included in this study, 34.9% had HER2-low breast cancer and 65.1% had HER2-0 breast cancer. Among the 127 patients with pathological information for both primary and metastatic lesions, 39.4% had HER2-low primary lesions and 43.3% had HER2-low metastatic lesions. A pooled analysis of 2310 HER2-negative breast cancer patients from four prospective neoadjuvant clinical trials showed that 34% of patients in the HoR-negative subgroup had HER2-low breast cancer, consistent with our study results (18). However, the patients in this study were early breast cancer patients from different clinical trials, leading to unavoidable heterogeneity, and they were from Western countries, so the results may not be generalizable to the Chinese population. A study involving 1433 Chinese HER2-low MBC patients showed that 35.3% of patients in the HoR-negative subgroup had HER2-low breast cancer, also consistent with our results (29). However, this study assessed HER2 IHC status based on primary tumors, and most patients did not undergo re-biopsy of metastatic lesions, so the possibility of discordant HER2 status cannot be excluded. Our results, reporting the proportions of HER2-low, HER2 IHC 0, HER2 IHC 1+, and HER2 IHC 2+ in primary and metastatic lesions in the mTNBC population, validate and supplement previous research. Additionally, being a single-center study, we avoided inter-laboratory heterogeneity in the HER2 status assessment.

Our study results indicate that among mTNBC patients, those with HER2-low breast cancer were older and had a lower proportion of premenopausal patients compared to HER2-0 patients. Previous studies on the clinicopathological characteristics of HER2-low breast cancer have shown inconsistent results. A pooled analysis revealed that HER2-low patients were older (*p* = 0.036), had a lower proportion of grade III tumors (*p* < 0.0001), and had lower Ki-67 status (*p* < 0.0001) (18). However, another study focusing on advanced breast cancer found no significant differences in demographic or baseline disease characteristics between HER2-low and HER2-0 groups, regardless of HoR status (30). A study of Chinese HER2-low MBC patients found that in the HoR-negative subgroup, a higher proportion of HER2-low patients were initially diagnosed with stage IV tumors (*p* = 0.006) (29). Therefore, based on the current evidence, it cannot be concluded that HER2-low breast cancer is more aggressive.

In this study, we also observed significant discordance in HER2-low status between primary and corresponding metastatic lesions (K=0.34, 95% CI 0.17-0.50, *p*<0.001), primarily characterized by a transition from HER2-0 to HER2-low. The baseline clinicopathological characteristics of patients with concordant HER2 status and those with discordant HER2 status were similar, and no potential factors influencing the change in HER2 status between primary and metastatic lesions were identified. In a study on the evolution of HER2-low status between primary and metastatic lesions, significant discordance was observed in the TNBC subgroup, with 31% of patients transitioning from HER2-0 to HER2-low, and 50% transitioning from HER2-low to HER2-0 (31). Another study involving 547 patients showed an inconsistency rate of 36.7% in HER2 status among TNBC patients, with 13.9% transitioning from HER2-0 to HER2-low, and 16.5% transitioning from HER2-low to HER2-0 (32). These findings highlight the dynamic nature of HER2 expression, particularly the transition between HER2-0 and HER2-low statuses during disease progression. The discordance in HER2 status observed in this study may be attributed to several factors. Preclinical studies have shown that anticancer treatments can lead to upregulation of HER2 expression in tumor cells, possibly as a resistance mechanism to the treatments received. This phenomenon has been observed in endocrine therapy (33), chemotherapy (34), and anti-HER2 therapy (35). A study evaluating the discrepancy rates between primary breast cancer and corresponding bone metastases found that previous anti-HER2 therapy was an independent risk factor for changes in HER2 status (36). Another study involving 549 patients reported that 19.8% of patients experienced changes in HER2 status after receiving neoadjuvant anti-HER2 therapy (*p*=0.009) (37), suggesting that prior anti-HER2 treatment was associated with changes in HER2 status. However, since all patients in this study were mTNBC patients and none had a history of prior anti-HER2 therapy, this conclusion could not be verified. HER2-related testing techniques might also contribute to the inconsistency in HER2 expression (8). Studies have shown poor interobserver agreement in the interpretation of HER2 IHC scores of 1+ and 2+ cases (38). Additionally, HER2 heterogeneity, which refers to the variability in HER2 expression or amplification within different regions of the same tumor or across different sites and times in the same patient, can also lead to discordance. A study of 96 patients comparing HER2 expression in three different regions of samples using tissue microarrays found greater heterogeneity in IHC 1+ and IHC 2+ patients compared to IHC 3+ patients (39). A retrospective analysis of HER2 IHC staining patterns in 281 breast cancer patients showed that various patterns of heterogeneity appeared more frequently in IHC1+ than in IHC2+ (40). The heterogeneity in HER2 expression in breast cancer may result in discrepancies between core needle biopsy (CNB) and surgical excision biopsy (SEB) samples. Several studies have investigated the concordance between CNB and SEB, with HER2 status agreement rates ranging from 56% to 98.3% (41, 42). Treatment decisions for MBC depend on ER, PR, and HER2 status. Some guidelines recommend a biopsy of metastatic lesions to reassess receptor status (43–45). Considering the promising efficacy of novel anti-HER2 drugs in HER2-low MBC (46, 47), and the discordance in HER2 status between primary and metastatic lesions observed in this study, retesting HER2 status in metastatic lesions is valuable.

In addition to analyzing changes in HER2 status, we also assessed the impact of HER2-low status on prognosis. Our results showed no significant differences in PFS and OS between HER2-low and HER2-0 patients undergoing first-line platinum-based chemotherapy in mTNBC patients (mPFS: 7.43 vs. 8.30 months, p=0.389; mOS: 25.37 vs. 26.63 months, p=0.907). Despite the limited sample size of our study, these findings are consistent with previous similar studies. Currently, HER2-low MBC is generally treated as HER2-negative MBC in clinical practice, with limited options for subsequent treatments. Trastuzumab was once considered potentially effective in HER2-low early breast cancer (16, 17), but the NSABP B-47 study indicated that adding trastuzumab to standard adjuvant therapy did not confer survival benefits to HER2-low patients (10). Several retrospective studies have indicated that HER2-low status does not impact prognosis (23, 24, 48). In the study by Agostinetto et al., no significant differences in PFS and OS were found between patients with HER2-low breast cancer and those with HER2+/HER2-0 breast cancer (23). Similarly, studies by Horisawa et al. and Schettini et al. comparing the prognosis of HER2-low and HER2-0 patients found no significant differences in outcomes, regardless of HoR status (12, 49), which aligns with our findings. However, some retrospective studies have also observed opposite findings. Carsten et al. reported that HER2-low breast cancer patients had a better prognosis compared to HER2-0 breast cancer patients, particularly among HoR-negative patients (3-year DFS: 84.5% *vs.* 74.4%, *p*=0.016) (18). These inconsistent results make the prognostic value of HER2-low an unresolved issue that requires further research.

In a previous study conducted by our research group, we explored the clinical characteristics, HER2 expression in primary and metastatic lesions, and therapeutic efficacy of HoR-positive/HER2-low breast cancer patients (50). The study found that 54.37% of HoR-positive MBC patients had HER2-low expression. The clinical characteristics were similar between HER2-low and HER2-0 patients, and there was no significant difference in PFS between the two groups receiving endocrine therapy (mPFS: 8.05 *vs* 10.12 months, *p*=0.114). However, in patients receiving chemotherapy, the PFS was significantly shorter in the HER2-low group compared to the HER2-0 group (mPFS: 8.64 *vs* 9.04 months, *p*=0.027). Interestingly, for patients receiving first-line platinum-based chemotherapy in the advanced setting, there was no significant difference in PFS between the HER2-low and HER2-0 groups (mPFS: 13.44 *vs* 12.12 months, *p*=0.332). These findings piqued our interest in exploring the efficacy of chemotherapy in HoR-negative/HER2-low patients, which led to the current study.

Most importantly, we found that patients with discordant HER2 status had better responses to first-line platinum-based chemotherapy compared to those with concordant HER2 status. Additionally, patients transitioning from HER2-0 to HER2-low had significantly longer PFS compared to those with concordant HER2-0 status. Similar results were observed in our previous study evaluating the clinical characteristics and treatment efficacy of HoR-positive/HER2-low MBC. HoR-positive patients whose HER2 status transitioned from 0 to low showed longer PFS when receiving first- or second-line chemotherapy compared to those with concordant HER2-low (*p*=0.048). However, the concordance of HER2 status did not significantly impact PFS for first- or second-line endocrine therapy or chemotherapy (50). In a study by Miglietta et al., the possible survival differences according to HER2-low evolution from primary to recurrent breast cancer were exploratively analyzed, and no significant impact on post-recurrence survival (PRS) of either HER2-low concordance or discordance was observed (32). A study by Elisa et al. involving 74 patients showed that those with a loss of HER2 expression in metastatic lesions had shorter PFS (*p*=0.03) and OS (*p*=0.001) compared to patients whose HER2 expression remained unchanged. These studies indicate that the impact of HER2 status evolution on prognosis remains controversial.

Our study has several limitations. Firstly, as a single-center study, the patients were derived from previous prospective studies conducted at our center. This led to more homogeneous treatment regimens, which may not fully reflect the diverse treatment patterns seen in real-world settings. However, considering that platinum-based chemotherapy remains a standard treatment for mTNBC, we believe the findings still hold clinical relevance. Secondly, some patients included in this study underwent pathological examinations at other hospitals. However, the majority of patients had their pathology re-evaluated by experienced pathologists at our center after entering our clinical trial, aiming to minimize inter-laboratory heterogeneity in HER2 testing. Thirdly, although our study includes extensive pathological and histological data, genomic information on HER2-low patients was not included. We plan to conduct further genomic analysis on HER2-low patients in future studies to provide more comprehensive biological insights.

## Conclusion

In conclusion, we found that in mTNBC patients, HER2-low patients had similar responses to chemotherapy as HER2-0 patients. There was a significant discordance in HER2 status between the primary and metastatic lesions of breast cancer. Patients with discordant HER2 status had better responses to first-line platinum-based chemotherapy. Therefore, for patients with HER2-0 primary lesions, re-evaluation of the HER2 status in metastatic lesions through biopsy may offer new treatment opportunities.

## Data Availability

The raw data supporting the conclusions of this article will be made available by the authors, without undue reservation.
