# Supplementary material for: Clinicopathological characteristics, evolution, and treatment outcomes of hormone receptor-negative/HER2-low metastatic breast cancer: a pooled analysis of individual patient data from three prospective clinical trials
Source: Front Endocrinol (Lausanne). 2024 Nov 21;15:1449278. doi: 10.3389/fendo.2024.1449278 (PMC11617163; doi:10.3389/fendo.2024.1449278)
Supplement: Supplementary file 1 [file DataSheet1.docx]

**Table S1.** **Key Design and Characteristics of the Three Prospective Clinical Trials Included in the Pooled Analysis.**

| **Study ID** | **Design** | **Sample Size** | **Key Inclusion Criteria** | **Chemotherapy Line** | **Treatment Regimen** | **Outcome Measures** |
| --- | --- | --- | --- | --- | --- | --- |
| **NCT00601159** | Phase II, Randomized Controlled Trial | 64 | 1. mTNBC: ER-negative (IHC <10%) , PR-negative (IHC <10%)^1^ and HER2-negative (IHC 0; FISH confirmation for IHC 1+ or 2+ samples) 2. No prior chemotherapy for metastatic disease or locally recurrent breast cancer^2^ | 1st line | GP | PFS, OS |
| **NCT02546934**  **(GAP)** | Phase III, Randomized Open-label Trial | 254 | 1. mTNBC: ER-negative and PR-negative (IHC<1%)^1^, HER2-negative (IHC 0-1+ or FISH ratio <2.0 if IHC 2+) 2. No prior chemotherapy or targeted therapy for metastatic disease^2^ | 1st line | GP *vs* AP | PFS, ORR, OS |
| **NCT02341911**  **(GPGC)** | Phase II, Randomized Open-label Trial | 146 | 1. Triple-negative: ER <1%, PR <1% and HER2-negative (IHC 0; FISH confirmation for IHC 2+, optional for IHC 1+) 2. No prior chemotherapy for metastatic disease^2^ | 1st line | GP *vs* GC | PFS, ORR, OS |

mTNBC: Metastatic triple-negative breast cancer; ER: Estrogen receptor; PR: Progesterone receptor; HER2: Human epidermal growth factor receptor 2; IHC: Immunohistochemistry; FISH: Fluorescence in situ hybridization; PFS: Progression-free survival; ORR: Objective response rate; OS: Overall survival.

1. ER-negative and PR-negative were defined slightly differently across the studies: in NCT00601159, they were defined as IHC <10%, while in NCT02546934 and NCT02341911, they were defined as IHC <1%.
2. Chemotherapy Line refers to the line of chemotherapy the patients received. In all studies, patients were receiving their first line of chemotherapy for metastatic disease.
3. Treatment Regimen: GP: Cisplatin combined with Gemcitabine; AP: Nab-paclitaxel combined with Cisplatin; GC: Gemcitabine combined with Carboplatin.

**Table S2. Definition of HER2 Status.**

| **HER2 Status** | **IHC** | **ISH** |
| --- | --- | --- |
| HER2- negative |  |  |
| Zero Expression (HER2-0) | 0 | Negative |
| Low Expression (HER2-low) | 2+ | Negative |
|  | 1+ | Negative |
|  | 1+ | Unknown |
| HER2- positive | 3+ | Positive |
|  | 2+ | Positive |

IHC, Immunohistochemistry; ISH, *In Situ* Hybridization.


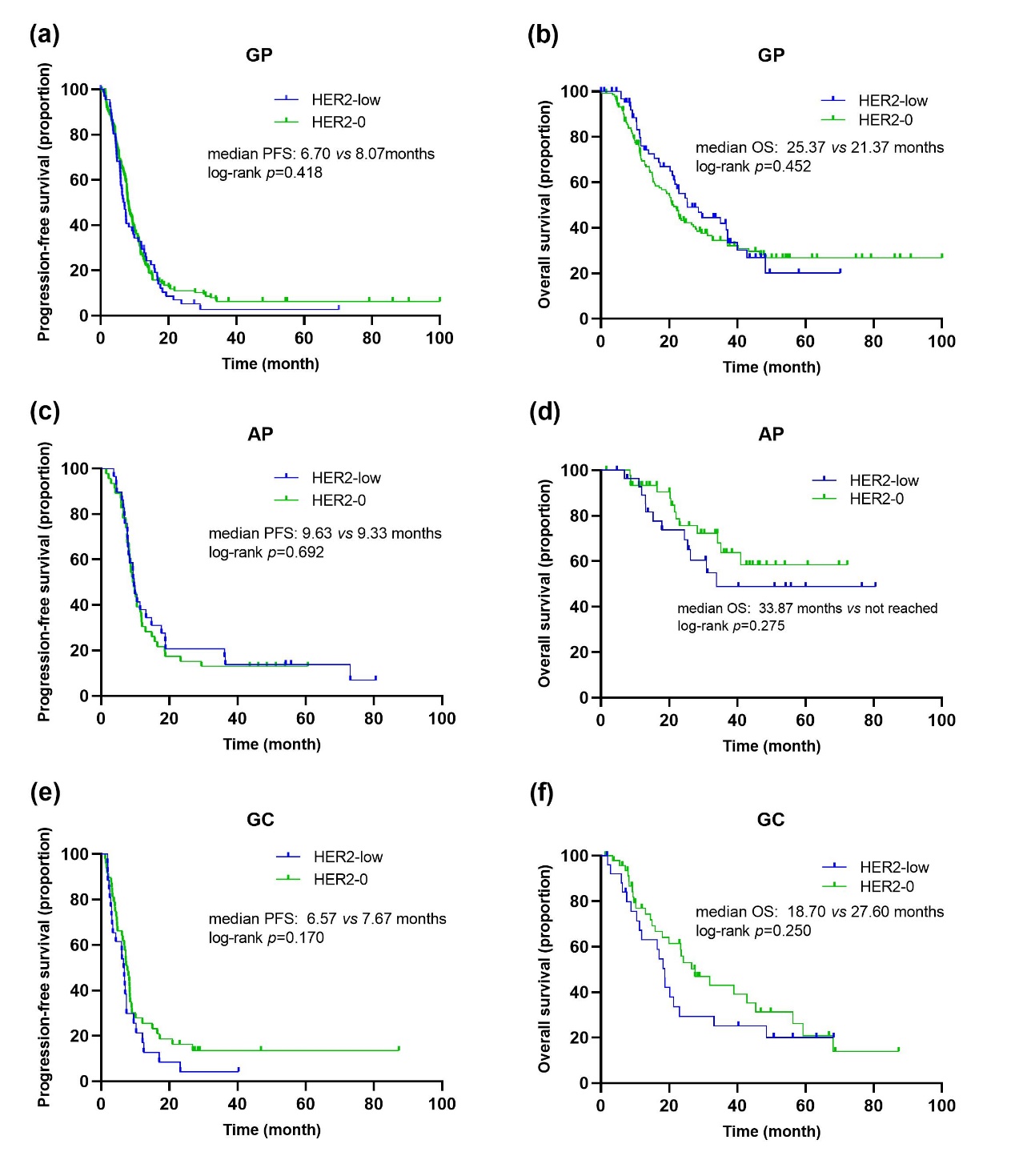


**Figure S1.** Kaplan-Meier curves of PFS and OS during first-line platinum-based chemotherapy, stratified by different treatment regimens. **(a)** PFS in HER2-low *vs.* HER2-0 patients receiving gemcitabine combined with cisplatin (GP). **(b)** OS in HER2-low *vs.* HER2-0 patients receiving GP. **(c)** PFS in HER2-low *vs.* HER2-0 patients receiving nab-paclitaxel combined with cisplatin (AP). **(d)** OS in HER2-low *vs.* HER2-0 patients receiving AP. **(e)** PFS in HER2-low *vs.* HER2-0 patients receiving gemcitabine combined with carboplatin (GC). **(f)** OS in HER2-low *vs.* HER2-0 patients receiving GC.


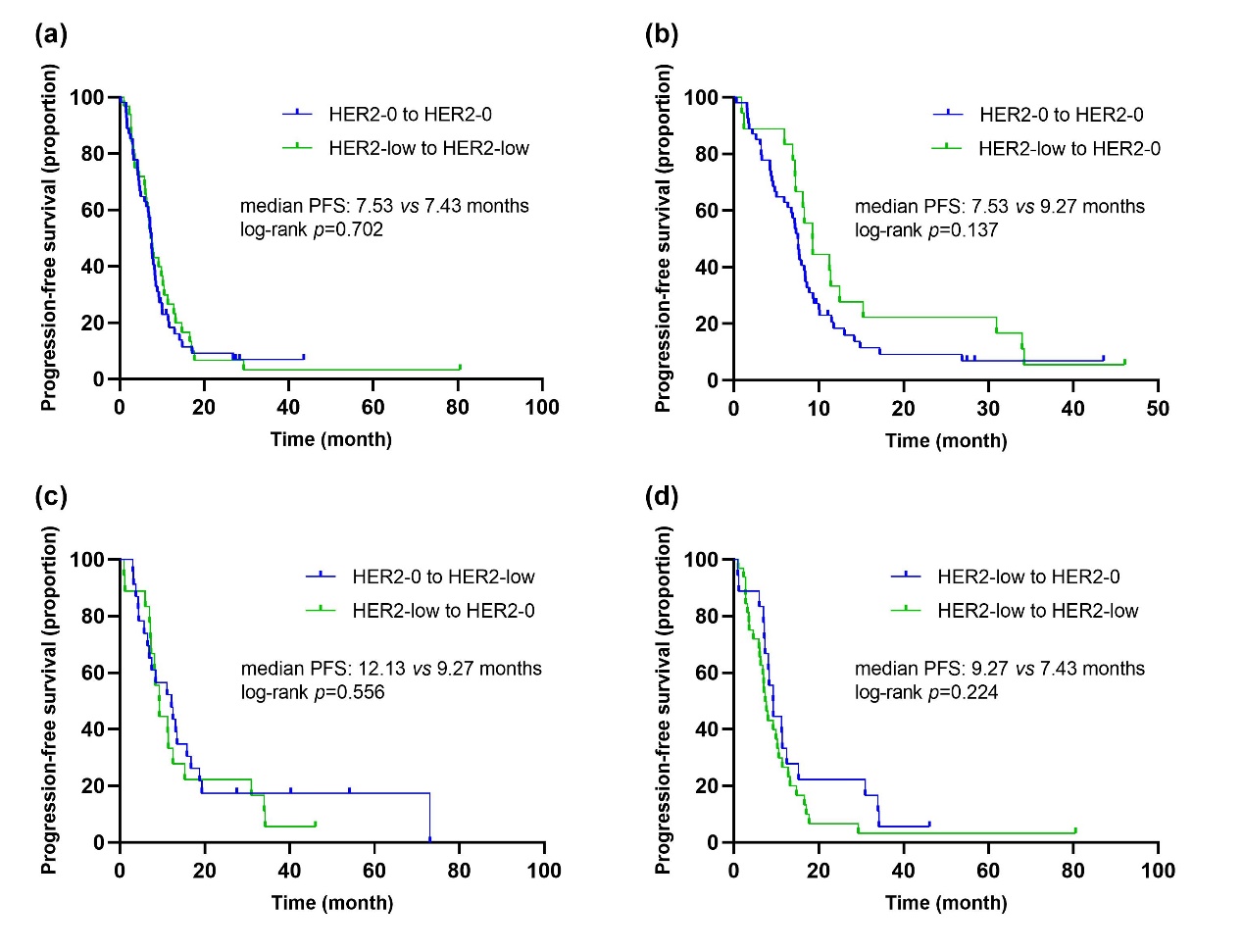


**Figure S2.** Kaplan-Meier curves of PFS in the first-line platinum-based chemotherapy, stratified by changes in HER2 status from primary to metastatic breast cancer. **(a)** Comparison of PFS in patients with changes from HER2-low to HER2-low *vs.* HER2-0 to HER2-0. **(b)** Comparison of PFS in patients with changes from HER2-0 to HER2-0 *vs.* HER2-low to HER2-0. **(c)** Comparison of PFS in patients with changes from HER2-0 to HER2-low *vs.* HER2-low to HER2-0. **(d)** Comparison of PFS in patients with changes from HER2-low to HER2-0 *vs.* HER2-low to HER2-low.


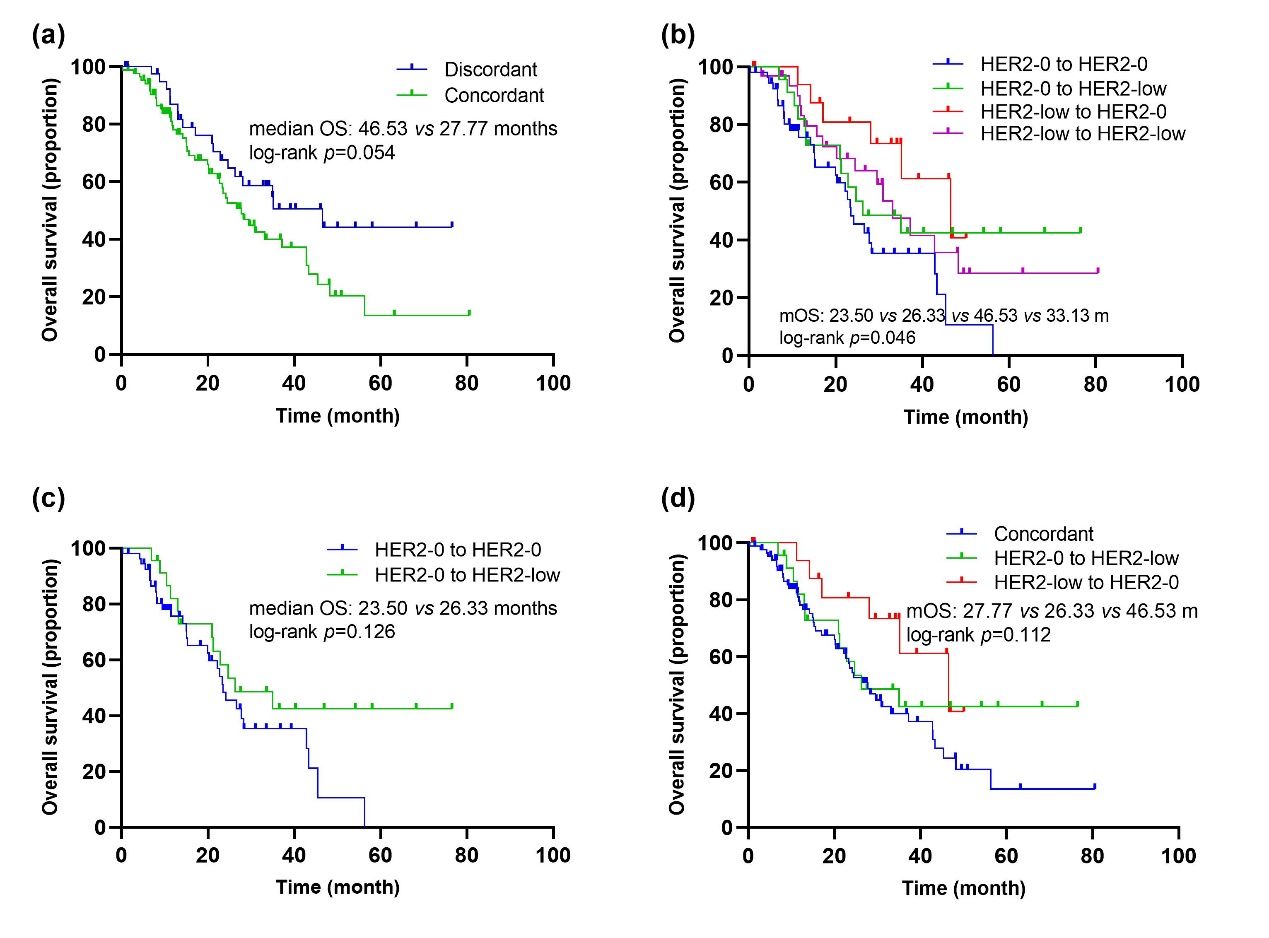


**Figure S3.** Kaplan–Meier curves of OS in the first-line platinum-based chemotherapy. **(a)** Comparison of OS in patients with concordant *vs.* discordant HER2 status between primary and metastasis breast cancer. **(b)** Comparison of OS in patients with different HER2 status transitions (HER2-0 to HER2-0 *vs.* HER2-0 to HER2-low *vs.* HER2-low to HER2-0 *vs.* HER2-low to HER2-low). **(c)** Comparison of OS in patients with different HER2 status transitions (HER2-0 to HER2-0 *vs.* HER2-0 to HER2-low). **(d)** Comparison of OS in patients with different HER2 status transitions (concordant HER2 status *vs.* HER2-0 to HER2-low *vs.* HER2-low to HER2-0).


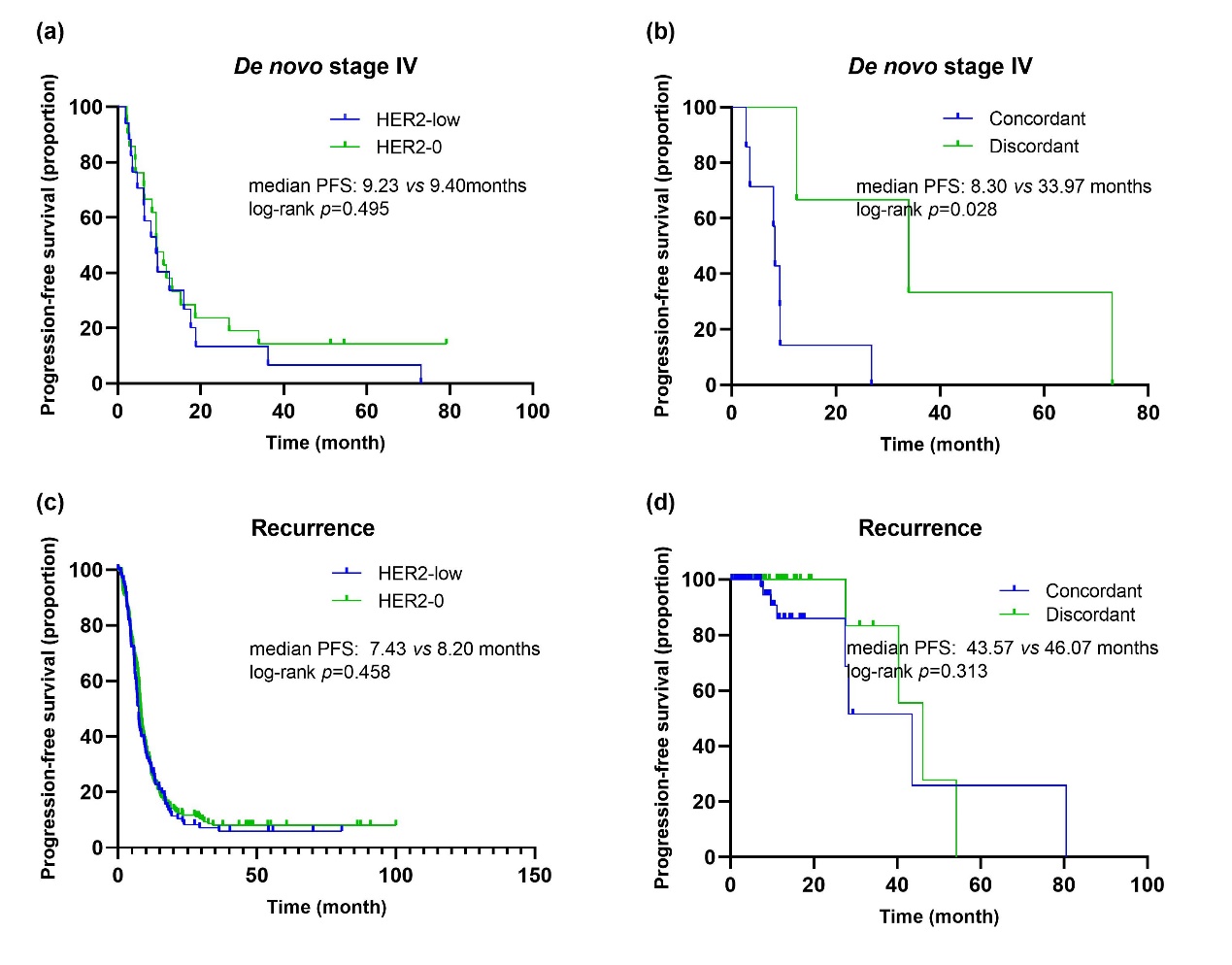


**Figure S4.** Kaplan–Meier curves of PFS in the first-line platinum-based chemotherapy. **(a)** Comparison of PFS in *De novo* stage IV patients with HER2-low and HER2-0. **(b)** Comparison of PFS in *De novo* stage IV patients with concordant *vs.* discordant HER2 status between primary and metastasis breast cancer. **(c)** Comparison of PFS in recurrent disease patients with HER2-low and HER2-0. **(d)** Comparison of PFS in recurrent disease patients with concordant *vs.* discordant HER2 status between primary and metastasis breast cancer.


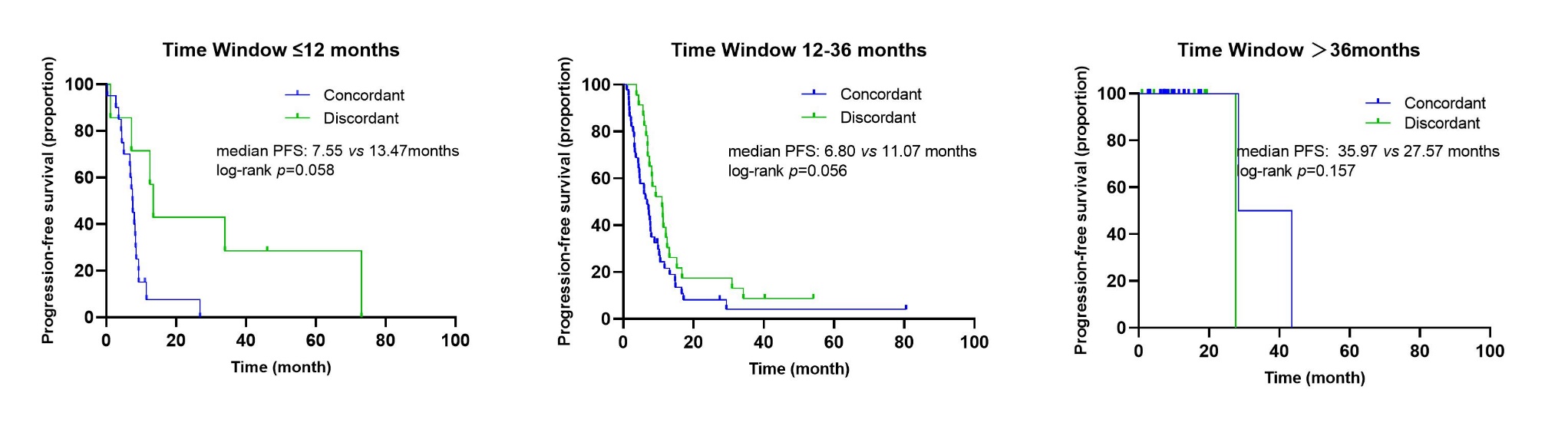


**Figure S5.** Kaplan–Meier curves of PFS in the first-line platinum-based chemotherapy. Comparison of PFS between patients with concordant *vs.* discordant HER2 status between primary and metastasis breast cancer, **(a)** in the ≤ 12 months time window subgroup. **(b)** in the 12-36 months time window subgroup. **(c)** in the > 36 months time window subgroup.

Time window: the time interval between the pathological reports of the primary and metastatic sites.
